# Development, psychometric evaluation and cognitive debriefing of the rheumatoid arthritis symptom and impact questionnaire (RASIQ)

**DOI:** 10.1186/s41687-021-00400-3

**Published:** 2021-12-11

**Authors:** Brandon Becker, Marguerite Bracher, Deven Chauhan, Regina Rendas-Baum, Xiaochen Lin, Kimberly Raymond, Meaghan O’Connor, Mark Kosinski

**Affiliations:** 1 GSK, Upper Providence, PA USA; 2 GSK, Stevenage, Hertfordshire UK; 3 GSK, Uxbridge, Middlesex UK; 4 QualityMetric Incorporated, LLC, Johnston, RI USA; 5 Present Address: Bristol-Myers Squibb, Lawrence Township, NJ USA

**Keywords:** Rheumatoid arthritis, Patient-reported outcome, RASIQ, Pain, Fatigue, Stiffness, Concept elicitation, Cognitive debriefing, Psychometric evaluation, Content validity

## Abstract

**Background:**

Rheumatoid arthritis (RA) is a chronic inflammatory disease often associated with persistent pain. There is a need for a patient-reported outcome measure (PROM) that is rooted in the patient experience and psychometrically validated. We describe the development of the Rheumatoid Arthritis Symptom and Impact Questionnaire (RASIQ), a novel PROM with potential to record key symptoms and impacts of RA with a 24-h recall period.

**Results:**

A literature review identified RA concepts that patients considered most important to their disease experience, including pain, fatigue, joint swelling and stiffness. From this, an initial item pool (33 items; 27 related to symptoms, 6 related to impacts) was developed with a recall period of 24 h. Two rheumatologists evaluated each item’s relevance, and the second version of the RASIQ was refined (29 items; 21 related to symptoms, 8 related to impacts). Next, three rounds of cognitive debriefing interviews were conducted with patients with RA (n = 15 overall). The RASIQ was revised to remove items deemed irrelevant or redundant, leaving 16 items measuring symptoms (joint pain, energy/tiredness, joint stiffness) and impacts (rest, sleep). A parallel series of semi-structured concept elicitation interviews (n = 30) facilitated the design of a conceptual model of RA symptoms, impacts and treatment experiences. Post-hoc comparison of the model with RASIQ revealed that all items selected were among the most important and relevant symptoms and impacts for patients. A final round of cognitive debriefing interviews (n = 12) confirmed that the final 16-item RASIQ was relevant and easy to understand, with no further changes recommended. Psychometric evaluation using data from two Phase II RA clinical trials confirmed a 3-factor structure, as well as the reliability and validity of the scale scores, and the ability of RASIQ to detect changes in symptoms and impacts when administered at specific study timepoints, using a 24-h recall period.

**Conclusions:**

RASIQ is a novel, 16-item PROM developed with substantial patient input. Results from concept elicitation, cognitive debriefing, and psychometric evaluation confirmed the validity of the instrument, which has the potential to measure symptoms and impacts through a 24-h recall period and complement existing disease activity instruments with longer recall periods.

**Supplementary Information:**

The online version contains supplementary material available at 10.1186/s41687-021-00400-3.

## Background

Rheumatoid arthritis (RA) is a chronic and inflammatory autoimmune disease that predominantly affects the joints, but can also include extra-articular manifestations or systemic comorbidities [1]. RA is known to have substantial impacts to patients’ health-related quality of life (HRQoL), affecting both physical and psychological domains [2, 3].

A number of disease-modifying antirheumatic drugs (DMARDs) are now available, including biologics and targeted synthetic agents [4]. However, patients often continue to experience impairments in their HRQoL and unmanageable pain when receiving treatment [3, 5]. A survey of patients with RA in the US who had received DMARD treatment found that pain continued to have a moderate-to-severe impact in three-quarters of respondents, and over half identified pain as the most bothersome symptom of RA [6]. Patients often report that their symptoms can resolve or flare up suddenly, and this uncertainty impacts negatively on their daily lives [7]. Additionally, RA is associated with a substantial economic burden, arising from direct medical-related costs as well as indirect costs, such as reduced productivity at work [8]. These factors demonstrate the importance of capturing patient perspectives of RA symptoms and impacts in clinical trials, in addition to assessing traditional composite endpoints.

A variety of patient-reported outcome measures (PROMs) have been used in RA clinical trials to provide insights into patient perspectives [9, 10]. The US Food and Drug Administration (FDA) has published guidance on the development and validation of such PROMs, including a recommendation for patient input early in the development process [11]. However, many PROMs used to assess RA were not developed according to these guidelines and did not involve patients in their development or validation [12]. Furthermore, some PROMs used in RA include complex concepts and use only a single item for each, potentially limiting understanding of RA symptoms and impacts, and the capture of treatment-related changes [9, 13]. For example, the Rheumatoid Arthritis Impact of Disease (RAID) evaluates 7 RA impact domains using a single numerical rating scale (NRS) for each [14], meaning that only the frequency or severity of each impact is considered, rather than both. In addition, many existing PROMs (such as RAID [14], and Routine Assessment of Patient Index Data 3 [RAPID3] [15]) have recall periods of 1 week [9, 14, 15], and may not detect short-term changes in symptoms or impacts.

This paper describes the development and validation of the Rheumatoid Arthritis Symptom and Impact Questionnaire (RASIQ), a PROM designed to record relevant symptoms of RA and their impacts on patients, which, with further development, has the potential to be used as a daily diary in clinical trials. The development and validation process involved significant patient input to ensure inclusion of the concepts most important to patients.

## Methods

### Phase I: Literature review

**Fig. 1:**
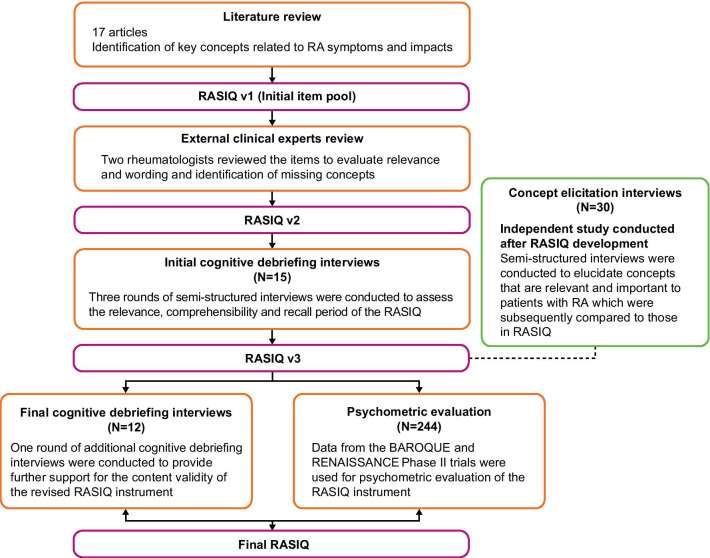
Overview of the RASIQ development process. PROM, patient-reported outcome measure; RA, rheumatoid arthritis; RASIQ, Rheumatoid Arthritis Symptom and Impact Questionnaire

This research comprised four phases, summarized in Fig. 1. In Phase I, a literature review was undertaken to identify key symptoms and impacts of RA, and evaluate the PROMs currently used in RA trials. Emphasis was placed on identifying symptoms or impacts that fluctuate daily, as these may not be adequately captured with current instruments. Searches were conducted in October 2013 using MEDLINE (2001–2013), EMBASE (2001–2013) and PsycINFO (2001–2013), supplemented by additional web searches of the Outcome Measures in Rheumatology (OMERACT) and Critical Path Institute (C-Path) Patient Reported Outcome Consortium websites. Search terms relating to RA, symptoms of RA and qualitative methodology (Additional file 1: Supplementary Methods) were used to identify articles. Studies were included if they related to adult patients (aged ≥ 18 years) with moderate-to-severe RA.

For the review of existing patient-reported outcome (PRO) symptom instruments, searches were conducted using MEDLINE (2001–2013), EMBASE (2001–2013), and supplemented with a search of the clinicaltrials.gov registry, the FDA and European Medicines Agency (EMA) websites, and the PROLabels Database [16]. Search terms relating to PROMs and measurement scales (Additional file 1: Supplementary Methods) were employed to identify PROMs that have been used in patients with RA. Studies mentioning instrument use in an RA patient population were included.

An initial item pool was created based on findings from the literature review, termed RASIQ v1.

### Phase II: Clinical expert review

In Phase II (Fig. 1), two rheumatologists reviewed the RASIQ v1 by evaluating the relevance of each item, suitability of the wording, appropriateness of the recall period, and identifying any content gaps. The agreed items were codified in the RASIQ v2.

### Phase III: Initial cognitive debriefing interviews

Phase III consisted of three rounds of cognitive debriefing interviews with patients with RA (Fig. 1). Patients were recruited from two clinical sites in the US: Coastal Clinical Research, Alabama and Health Research of Hampton Roads, Virginia. Potential interview participants were identified by clinical staff through the sites’ patient registers and medical chart review and were invited to participate, subject to confirmation that patients met the eligibility criteria and provided written informed consent. The purpose of the interviews was to evaluate the relevance, comprehensiveness, and comprehensibility of the items and response options in RASIQ v2. Between each round of interviews, the RASIQ v2 was revised based on the participant feedback. The resulting items were codified in the RASIQ v3.

Participants were eligible if they were ≥ 18 years of age with a diagnosis of RA for ≥ 1 year, and currently receiving methotrexate at a stable dose (Additional file 1: Table S1). Cognitive debriefing interviews were conducted in-person with a trained investigator using a semi-structured interview guide and a think-aloud method. In the think-aloud method, the participant completes the instrument by reading aloud all elements (instructions, items, response options) and vocalizing their decision-making processes and answers. This is a standardized approach to cognitive debriefing: it provides insights into the participant’s comprehension of each element; the rationale for selecting a particular response; and, as the investigator remains silent throughout the process, reduces the chances of the investigator introducing bias into the patients’ responses [17]. At the start of their interview, participants were asked to complete RASIQ v2; for questions on symptom severity, patients were asked to consider each symptom at its worst during the past 24 h and to rate the symptom severity on a mixture of Likert frequency and severity scales and NRS severity scales. Three rounds of interviews took place to allow for feedback from earlier interviews to be incorporated in subsequent interviews. Cognitive debriefing data was analyzed as a whole and on an item-by-item basis, to identify items that presented cognitive challenges and to assess content validity.

### Phase IVa: Final cognitive debriefing interviews

The final phase of the RASIQ development included a set of cognitive debriefing interviews with patients with RA, and psychometric evaluation, conducted concurrently (Fig. 1).

The cognitive debriefing interviews aimed to assess the comprehensiveness, comprehensibility, and relevance of the RASIQ v3. Patients in the US (Philadelphia and New York) were identified by QualityMetric and Schlesinger Group and provided written informed consent. Eligible participants were aged ≥ 18 years with a self-reported clinician diagnosis of moderate-to-severe RA for ≥ 2 years with ≥ 6 swollen and ≥ 6 tender joints, and had received DMARD and/or a biologic treatment for RA in the past 2 years; participants were also required to have experienced symptoms of RA in the past 7 days (Additional file 1: Table S1). A 60-min, one-on-one qualitative interview (GSK study 206981) was conducted with each participant either in-person or by phone by a trained qualitative researcher. Participants completed RASIQ v3 using the think-aloud method, during which the researcher took notes to capture observations and highlight areas for further inquiry. Following the think-aloud, participants answered specific, structured questions regarding the overall instrument, including relevance of the items, clarity of the instructions, the recall period, and each individual item and accompanying response options. For data analysis, audio recordings of all interviews were transcribed verbatim and coded for content related to each element of the RASIQ. Items were also assigned codes to designate whether the item was viewed as problematic or irrelevant by participants. Findings from the psychometric evaluation and final cognitive debriefing interviews informed the development of the final RASIQ PROM.

### Phase IVb: Psychometric evaluation

Psychometric evaluation assessed the measurement properties of the instrument and confirmed the items and structure for the final RASIQ. Data from two Phase II randomized, multicenter, double-blind, placebo-controlled trials assessing otilimab, an investigational anti–granulocyte–macrophage colony-stimulating factor (GM-CSF) monoclonal antibody, in patients with RA, RENAISSANCE (N = 39; GSK study 205180; NCT02799472) and BAROQUE (N = 222; GSK study 201755; NCT02504671), were used for psychometric evaluation of RASIQ v3. The study designs have been described previously [18, 19]; see Additional file 1: Table S1 for eligibility criteria. For RENAISSANCE, RASIQ v3 was completed on an electronic device using a 24-h recall by patients on the day of the scheduled clinic visit at each of the following timepoints: screening, baseline (n = 39), Weeks 1 (n = 36), 6 (data not used for this analysis), and 12 (n = 32), and at follow-up (data not used for this analysis). For BAROQUE, RASIQ v3 was completed on an electronic device using a 24-h recall by patients on the day of the scheduled clinic visit at each of the following timepoints: baseline (n = 202), Weeks 1 (n = 196), 12 (n = 192), 24 (n = 109), 36 (n = 53), and 52 (n = 42), and at follow-up (n = 41). Psychometric analysis was independent of the main clinical trial analyses and conducted post hoc with blind data.

Multi-trait analysis [20] was conducted in an iterative manner using data from baseline, Week 1 and Week 12 to evaluate the measurement model of RASIQ v3 and test the appropriateness of using a summated scoring algorithm (Additional file 1: Table S2 and accompanying text). Mokken scale analysis [21, 22] was conducted to determine whether the response options provided to the RASIQ were correctly ordered and monotonic. A cutoff of 0.03 was used for the item-scalability coefficient [23], and violations above this cutoff were tested for statistical significance. The test-scalability coefficient was also evaluated for the RASIQ as a whole, as well as for scales containing ≥ 3 items. It has been proposed that a scale should be considered weak if 0.3 ≤ *H* < 0.4, moderate if 0.4 ≤ *H* < 0.5, and strong if *H* > 0.5, where *H* is the test-scalability coefficient. Item-level convergent validity, discriminant validity, and internal consistency reliability were assessed.

Confirmatory and exploratory factor analyses (CFA and EFA, respectively) were used for further refinement of the measurement model and to evaluate the number of factors required to explain variation across the item set. CFA and EFA were replicated using baseline and Week 12 data, such that when EFA was conducted using the baseline data, CFA results from Week 12 data were considered, and vice versa. EFA was conducted using the maximum likelihood estimator and Geomin rotation, and a method previously described by Joreskog et al*.* was used to conduct an EFA in a CFA framework [24]. For each factor structure, goodness of fit was evaluated with the Comparative Fit Index, the Tucker–Lewis Index, and the root mean square error of approximation.

Cronbach’s alpha was used to evaluate the internal consistency reliability of each scale in RASIQ v3 [25], and the intraclass correlation coefficient (ICC) of the item and domain scores of the RASIQ was used to assess test–retest reliability [26]. Test–retest reliability was evaluated using data from a sample of RA patients (N = 25) who were “clinically stable” in each trial. Specifically, the stable sample consisted of patients from the 2 trials who did not show a change of 20% or greater in tender (28) and swollen (28) joint counts and any of the 5 remaining American College of Rheumatology (ACR) core set measures (Physician’s global assessment of disease activity (PhGA), patient’s global assessment of disease activity (PtGA), patient’s assessment of arthritis pain, Health Assessment Questionnaire-Disability Index (HAQ-DI), and C-reactive protein (CRP)) from baseline to Week 1, and had concurrent assessments of the RASIQ at both time points. Convergent and discriminant validity was evaluated using Pearson correlation between the scale scores from the RASIQ and scores from other PROMs.

Known-groups validity was evaluated by comparing the scale scores of the RASIQ between groups of participants with different levels of disease activity according to the following categories: Disease activity score-28 (DAS28) [27] was used to split participants into four groups: disease remission (DAS28 ≤ 2.6), low disease activity (2.6 < DAS28 ≤ 3.2), moderate disease activity (3.2 < DAS28 ≤ 5.1) and high disease activity (DAS28 > 5.1); PhGA and PtGA were used to split participants into three groups: no to mild disease activity (PhGA/PtGA ≤ 40), moderate disease activity (40 < PhGA/PtGA ≤ 70) and high disease activity (PhGA/PtGA > 70); Patient’s assessment of arthritis pain (measured using a validated visual analog scale [VAS] scale [28]) was used to split participants into three groups: no pain to mild pain (VAS ≤ 40), moderate pain (40 < VAS ≤ 70) and severe pain (VAS > 70). It was expected that changes in RASIQ would be correlated with changes in these measures with correlation coefficients ≥|0.30|.

To assess ability of the RASIQ to detect change in RA, correlations were computed between change from baseline to Week 12 in RASIQ score and changes in RA disease measures: DAS28, PhGA, PtGA, patient’s assessment of arthritis pain, tender and swollen joint count, and HAQ-DI.

### Concept elicitation interviews

In parallel with the development phases described above, a series of concept elicitation interviews with a different group of patients with RA were conducted (Fig. 1). These interviews did not directly assess RASIQ; the aim was to explore the symptoms, impacts, and treatment experience of RA and, in doing so, evaluate the relevance of the items included in the RASIQ.

Patients were recruited via the assistance of MedQuest Global, a recruitment agency, from four different geographic locations in the US: Baltimore, Maryland; Chicago, Illinois; New Orleans, Louisiana; St Louis, Missouri. Participants were recruited via a combination of six general practitioners and eight rheumatologists, and patients provided written informed consent. Eligible participants were ≥ 18 years of age with a diagnosis of RA for ≥ 6 months, ≥ 4 swollen and tender joints, and inadequate response to DMARDs (Additional file 1: Table S1). For concept elicitation, 60-min, semi-structured telephone interviews were conducted in the US by Adelphi Values (Macclesfield, Cheshire, UK). Two rounds of interviews were carried out so modifications could be made to the interview guide after round one. The interview guide was used by trained researchers, and included a series of broad, open-ended questions designed to elicit information about the participant symptom and treatment experiences, and to allow for concepts to be mentioned spontaneously by participants. Follow-up questions and prompts ensured that each topic could be fully explored.

For concept elicitation data analysis, all interviews were audio recorded and transcribed verbatim; qualitative analysis of transcripts was conducted with ATLAS.ti (ATLAS.ti Scientific Software Development GmbH, Berlin, Germany) using thematic analysis methods. Transcripts were coded to identify groups of concepts reported by participants. Concept saturation was assessed to ensure that all important concepts had emerged and been identified. To evaluate whether saturation had been achieved, transcripts were divided chronologically into five equal groups of six participants in each, and the number of new concepts identified in each group were evaluated. Findings from the concept elicitation interviews were used to develop a conceptual model for RA.

## Results

### Literature search and initial item pool selection (Phase I)

**Fig. 2:**
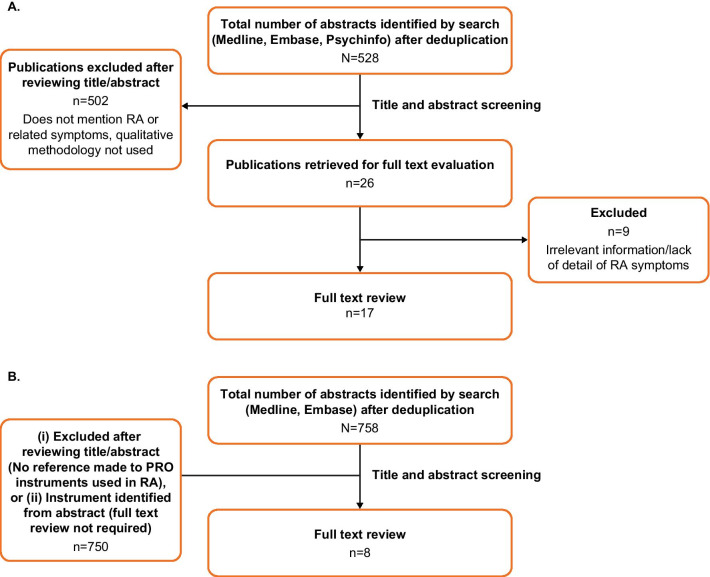
Literature search flow diagram (A) qualitative literature search (B) PRO instrument literature search. PRO, patient-reported outcome; RA, rheumatoid arthritis

The literature review included 17 articles describing focus groups or interviews with patients with RA (Fig. 2A; Additional file 1: Table S3). From those articles, numerous symptoms and impacts were identified as important to the experience of RA (Table 1). Pain, chiefly in the joints, was most frequently reported and rated as the most important symptom by patients with RA in several studies. Pain severity was also found to vary frequently. Joint stiffness, particularly in the morning after lying down for several hours, was found to be an important symptom. Fatigue was also rated as an important symptom that impacts patients’ quality of life, daily activities and emotions. Other impacts to emotional well-being and quality of life were also reported, although these were difficult for patients to define.

**Table 1:** RA symptoms and impact concepts identified by literature review, and associated concept elicitation interview quotes

Concept (literature review)	Supporting participant quote (concept elicitation interviews)
Pain	*“It´s just a constant pain. I can´t say that it´s dull, because it´s more of an ache.”*
Fatigue	*“I'm very fatigued. It's like there's a day where I want to fight it and go through it, but my body's saying you're just too tired, so if that makes sense.”*
Sleep	*“I mean it´ll go to the point of me not sleeping because of the pain and the swelling and everything for two or three days before I sleep. I´ll be past the point of exhaustion probably.”*
Energy	*“Yeah, it’s sort of the illness … sometimes, for whatever the reason, it’s as if you have been running a marathon, you’re tired, and very usually after 8 pm, I don’t have any energy.”*
Stiffness	*“When I wake up in the morning … my joints are stiff initially.”*
Mobility	*“Movement is … limited as far as time and how long I can endure something.”*
Joint swelling	*“I experience kind of the swelling. It usually starts with swelling, and then it really gets kind of tender and, and feels a little bit warmer."*
Tender joint	*“…tenderness, um, almost like a sharp pain.”*
Muscle strength	*“I don’t feel that I have the strength in, in my right hand as well as I do [in] the left”*
Physical function	*“I can’t lift any groceries or I can’t lift laundry. I can’t do none of that anymore. I don’t do none of that. I mean it’s impacting me tremendously”*
Life enjoyment	*“It takes away your life of enjoyment with your family. You can’t stay out in parks a long time with them. You want to go to family gatherings, you want to go to amusement parks. Things like that I can’t do anymore. So it’s, it’s depressing. It’s hurting that I can’t be in the lives of my grandkids like I want to be because of my condition.”*
Relationships	*“I'm scared to be with— just find somebody that, uh, you know, to get married—because I can't take care of anybody now”*
Depression	*“When you’re living a painful life, you can’t be happy”*
Well-being	*“Not being able to do things I like to do, not being able to focus. Basically, it's like I feel worthless”*

The literature review also included eight articles (Fig. 2B) that described 21 RA-specific PROMs; 12 of these were used in at least one RA clinical trial. None of the identified PROMs covered all of the important symptoms and impacts identified by the literature review. Indeed, several of the existing instruments focus on only one aspect of RA (e.g. fatigue) and one instrument, the RAID, which covers the most relevant symptoms and impacts of the described PROMs (e.g. pain, fatigue and sleep), lacks coverage of others (e.g. joint stiffness) [29].

Based on evidence from the literature review, the initial item pool for the RASIQ v1 contained 27 items related to symptoms, and 6 items related to impacts. A recall period of 24 h was selected to consider the daily variations in RA symptoms, particularly tiredness and pain.

### Clinical expert review (Phase II)

The clinical experts who reviewed the RASIQ v1 made several recommendations for improvements to the instrument. The term ‘pain,’ was considered too broad, and was replaced by ‘joint pain’. Further, an additional item on pain at night was recommended for inclusion. An item on ‘lack of energy’ was added to the symptoms, and an item covering ‘loss of balance’ was re-categorized from a symptom to an impact. Items on ‘muscle stiffness’ and ‘early morning joint stiffness’ were added, and a ‘single joint tenderness’ item was removed due to concern about potential overlap with ‘joint pain’. As a result, RASIQ v2 contained 29 items across 8 concepts (symptoms: joint pain [7 items], tiredness/energy [6 items], joint stiffness [5 items], swelling [1 item], muscle weakness [2 items]; impacts: sleep [4 items], rest [2 items], loss of balance [2 items]). RASIQ v2 also comprised a mixture of Likert frequency and severity scales and NRS severity scales were used for items. The experts recommended keeping both scale types and evaluating them by cognitive debriefing.

### Initial cognitive debriefing interviews (Phase III)

Fifteen individuals with RA (female: n = 13 [87%]; mean [standard deviation, SD] age: 57 [9] years) participated in cognitive debriefing interviews. The mean (SD) time since RA diagnosis was 12.3 (7) years. All participants were receiving methotrexate treatment for RA at the time of the interview (Additional file 1: Table S4). Interviews were split into three rounds (round 1, n = 6; round 2, n = 4; round 3, n = 5). The results from each round of interviews were used to refine the instrument before the next round.

Across the three rounds of interviews, six concepts were identified as relatively unimportant or of unclear relevance to patients with RA: loss of balance, mental tiredness, muscle stiffness, muscle weakness, resting due to tiredness, and joint swelling. For example, in round 1, although 3 out of 6 participants identified joint swelling as one of the most important symptoms, five participants provided feedback that swelling was very closely related to or the same as pain. Furthermore, two participants found the response options for swelling difficult to use; either because they did not feel the concept of swollen joints was relevant, or because they found the response option labels too vague and difficult to quantify their joint swelling. As a result, items related to joint swelling were removed after round 1. Based on the negative patient feedback regarding the aforementioned six concepts, 15 items were removed. Several previously combined items were also split into two, such as ‘joint pain when sitting and lying’, and ‘joint pain while walking and standing’.

After each round of interviews, the wording of several items was revised to improve clarity. For example, the item ‘In the past 24 h, how severe was your joint pain when standing?’ was reworded to ‘In the past 24 h, how severe was your joint pain at its worst when in a standing position?’, as one participant felt the original item meant joint pain from standing after sitting or lying down. One participant consistently responded using a recall period of longer than 24 h across different questions. During the third round of interviews, participants reported that the items were mostly comprehensive, relevant, and easy to understand.

Based on the updates made following the interviews, RASIQ v3 (Additional file 1: Table S5) contained 16 items—13 of which measured symptoms (joint pain [7 items], energy/tiredness [2 items], joint stiffness [4 items]) and three of which measured impacts (rest [1 item], sleep [2 items]). Participants had mixed preferences for the format of response options, with some preferring the Likert scale and others preferring the NRS. As some participants found it difficult to use a Likert scale to rate pain severity, joint pain items were rated 1–10 on an NRS; a Likert scale was used for the remaining items, which were rated 1–5, aside from one item rated 1–6 (‘How long did the early morning stiffness in your joints last today’).

### Final cognitive debriefing interviews (Phase IVa)

Twelve individuals with RA (females: n = 8 [67%]; mean [range] age: 53 [23–82] years) participated in the final cognitive debriefing interviews with the RASIQ v3. Most participants (n = 7; 58%) had been diagnosed with RA for ≥ 10 years (Additional file 1: Table S6).

Overall, participants described the RASIQ as clear and easy to understand. The majority of participants (n = 8; 67%) reported that all items on joint pain, joint stiffness and energy/tiredness were relevant to their experience of RA, and almost all (n = 11; 92%) felt that the sleep and rest items were relevant. Two participants reported that lifting objects was not relevant based on their day-to-day demands or activities and, for two other participants, pain when lying down did not apply to their experience of RA.

Participants reported that the symptoms included in the RASIQ covered the breadth of their RA experience and they had no suggestions of additional symptoms for inclusion. Although most participants reported that all sleep, rest, joint pain, joint stiffness, and tiredness/energy impact items were relevant to their RA, some suggested gaps related to impacts, including difficulties in walking, standing, or lifting objects. These impacts were not added to the RASIQ scale as they were generally encompassed by the joint stiffness and joint pain items in the symptoms scale. Other perceived gaps in impacts were reported such as opening jars or turning door handles, exercising, and social participation; primarily due to pain or swelling caused by RA. These impacts were not added to the RASIQ due to its potential future use as a daily questionnaire focusing on concepts most likely to fluctuate on a 24-h basis. Some participants suggested using an alternative title for the ‘Impacts’ scale that more accurately reflects the specific item content.

All 12 participants reported that the recall period of 24 h was easy to understand and remember, and that the length of the instrument was appropriate and not overbearing. The response options for each of the symptom-related items (both NRS and Likert scales) were also considered suitable and understandable. No changes were recommended or made to the RASIQ v3 based on the findings from the cognitive debriefing.

### Psychometric evaluation (Phase IVb)

Psychometric evaluation was performed on RASIQ v3 (Additional file 1: Table S5). At this stage in development, the instrument used a scoring algorithm based on summation of items within each scale after recoding so that all items were scored in the same direction, followed by a transformation to a 0–100 scale. Mokken scale analysis indicated that all item-scalability coefficients were > 0.30 when considering the entire RASIQ, the joint pain items, the joint stiffness items and the impact items (Table 2), supporting the monotonicity of the RASIQ response scales and the use of summated scoring algorithm for subsequent analyses. Test-scalability coefficients were ≥ 0.50 at each time point, indicating that items within the proposed scales are strongly scalable, that is the ordering of persons according to the summated score adequately reflects the order of the latent trait.

**Table 2:** Mokken scale analysis at baseline, Week 1 and Week 12

Scale	Baseline		Week 1		Week 12	
	Item scalability coefficient	Test scalability coefficient	Item scalability coefficient	Test scalability coefficient	Item scalability coefficient	Test scalability coefficient
Common scale (16 items)	0.37–0.69	0.60	0.40–0.74	0.66	0.53–0.80	0.73
Joint pain (7 items)	0.77–0.82	0.79	0.82–0.87	0.84	0.86–0.89	0.88
Joint stiffness (4 items)	0.64–0.71	0.68	0.59–0.71	0.65	0.74–0.80	0.76
Impact (5 items)	0.44–0.56	0.50	0.50–0.58	0.55	0.65–0.71	0.68

**Fig. 3:**
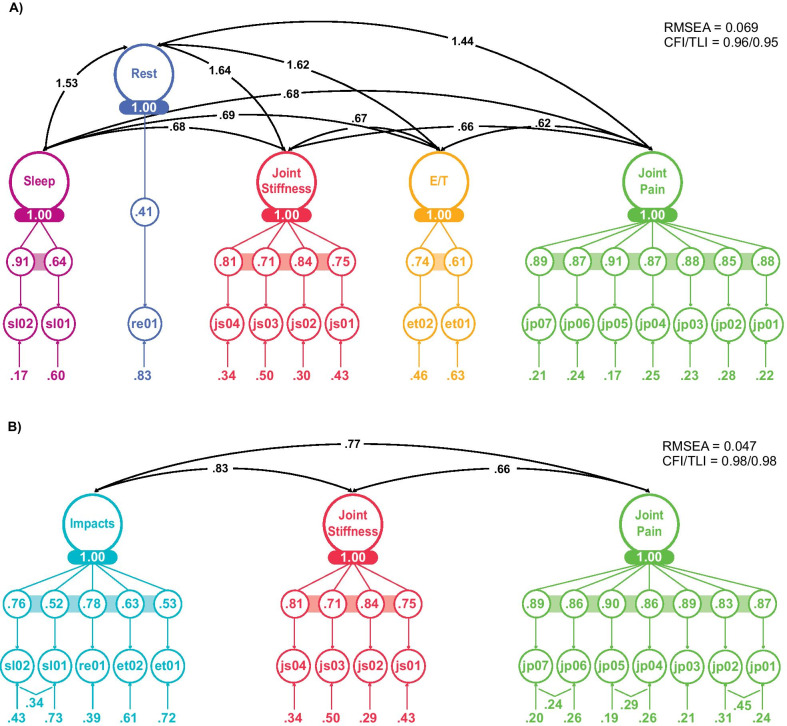
Estimated parameters for the 5-factor (A) and 3-factor (B) RASIQ structures. CFI, comparative fit index; RASIQ, Rheumatoid Arthritis Symptom and Impact Questionnaire; RMSEA, root mean square error of approximation; TLI, Tucker–Lewis index

The hypothesized measurement model used in this phase of development was a 5-factor structure comprising three symptom scales (Joint pain, Energy/Tiredness and Joint stiffness) and two impact scales (Rest and Sleep) (Fig. 3A). Based on findings from EFA, EFA in CFA and initial multi-trait analyses, alternate measurement structures were evaluated in a CFA framework, including a 3-factor alternative comprising two symptom scales (Joint pain and Joint stiffness) and a single impacts scale (Fig. 3B). While the 3-factor structure did not result in substantially better indices of global fit, it did result in slightly better statistical indices for some of the psychometric properties when compared with the 5-factor structure. We present results of both measurement models to document the similarities and psychometric differences between them.

When considering the 5-factor model, all scales (Joint pain, Energy/Tiredness, Joint stiffness, Rest and Sleep) had satisfactory internal consistency reliability (Cronbach’s alpha ≥ 0.7), with the exception of the Energy/Tiredness scale at baseline (Cronbach’s alpha = 0.62). For the 3-factor model, all Cronbach’s alpha coefficients were ≥ 0.80. In addition, only the Energy/Tiredness scale (ICC 0.55) and the rest item (ICC 0.60) under the 5-factor model were associated with lower values of test–retest reliability; all other scales had good test–retest reliability (ICC ≥ 0.7). Individual items within each scale were correlated (all ρ ≥ 0.40) with the total summed score from the proposed RASIQ scale within both the 5-factor and 3-factor structures, suggesting good convergent validity. Most items (n = 15/16 across baseline, Week 1 and Week 12 data) had a stronger correlation with their proposed scale than with scales measuring other concepts, suggesting adequate item-discriminant validity; the only exception was one Energy/Tiredness item (‘In the past 24 h, how often have you felt physically tired?’), which correlated significantly more strongly with the Rest (0.48) and Sleep (0.47) scales than with the Energy/Tiredness scale (0.45) at baseline. Indeed, during the cognitive debriefing interviews, one participant felt that there was overlap between ‘energy’, ‘exhaustion’ and ‘physical tiredness’. Correlations with the validation measures, including patient assessments of pain, PhGA, PtGA and HAQ-DI, were generally in the expected direction and magnitude (|ρ|≥ 0.30). Slightly higher correlations were observed for the Impact scale (3-factor structure) when compared with the individual Sleep, Energy/Tiredness and Rest scales from the 5-factor structure, lending support for the alternative 3-factor measurement model.

For known-groups validity, scales within the 5-factor and 3-factor structures generally showed the ability to differentiate patients by RA severity, based on the PtGA and the patient’s assessment of arthritis pain (VAS). Only the Sleep scale (5-factor structure) failed to differentiate patients with moderate and low disease activity, when defined by ESR-based DAS28 using Week 12 data, whereas all other scales were able to distinguish patients with high, moderate or low disease activity at Week 12 (Additional file 1: Table S7). Analyses based on PhGA indicated that the RASIQ scales were generally able to distinguish between groups; however, the Energy/Tiredness, Impact and Sleep scales were unable to distinguish moderate and severe PhGA groups (Additional file 1: Table S7).

The ability of the RASIQ to detect changes in RA was also assessed (Additional file 1: Table S8). Change scores for all RASIQ scales (5- and 3-factor structures) from baseline to Week 12 were correlated in the expected direction, at or above a meaningful level (ρ ≥ 0.3) with tender joint count, DAS28, PhGA, PtGA, patient’s assessment of arthritis pain, and HAQ-DI. Similarly, scales were generally able to discriminate between patients with different levels of clinical response from baseline using ACR and DAS28 criteria.

As described above, the 3-factor measurement structure provided higher correlations for the impact scale than the 5-factor structure. Furthermore, the 3-factor structure aggregated items covering sleep, rest and energy/tiredness into a single scale, and presents advantages for measurement and clinical trial implementation. As such, the 3-factor structure was selected for the final instrument (Table 3).

**Table 3:** Conceptual framework for the final 3-factor RASIQ instrument

Scale	Item
Joint pain	Severity of joint pain when sitting (past 24 h)
	Severity of joint pain when lying down (past 24 h)
	Severity of joint pain when starting to move after being still (past 24 h)
	Severity of joint pain when walking (past 24 h)
	Severity of joint pain when standing (past 24 h)
	Severity of joint pain when lifting objects (past 24 h)
	Severity of joint pain at its worst (past 24 h)
Joint stiffness	Frequency of joint stiffness (past 24 h)
	Severity of early morning stiffness in joints (when woke up today)
	Duration of early morning stiffness in joints (today)
	Severity of joint stiffness in the day (past 24 h)
Impacts	Level of energy (past 24 h)
	Frequency of physical tiredness (past 24 h)
	Frequency of rest due to joint pain (past 24 h)
	Sleep quality (last night)
	Sleep disturbance due to joint pain (last night)

### Concept elicitation

Thirty individuals with RA (females: n = 19 [63%]; mean [range] age: 53 [21–80] years) participated in the concept elicitation interviews (Additional file 1: Table S9). Participants had been diagnosed with RA for an average of 9 years (range: 9 months to 40.5 years). Post-hoc comparison of the symptoms and impacts identified during concept elicitation with RASIQ revealed that all items included in the final RASIQ instrument were relevant to the patient experience of RA.

#### Symptoms

The symptoms reported by participants corresponded well with those identified in the literature review; relevant quotes for each concept are shown in Table 1. Concept saturation (i.e., the point in the interview process at which no new concepts arise) was achieved for all symptoms, suggesting that the sample size of 30 was adequate.

**Fig. 4:**
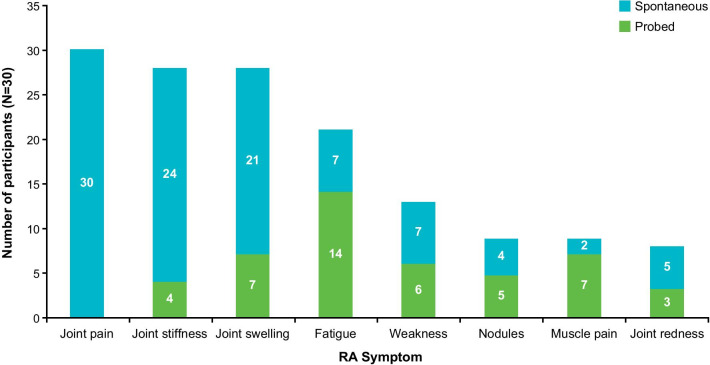
RA symptoms reported during concept elicitation, whether reported spontaneously or after probing. RA, rheumatoid arthritis

Participants reported eight key symptoms of RA during the concept elicitation interviews: joint pain, joint stiffness, joint swelling, fatigue, weakness, nodules, muscle pain, and joint redness (Fig. 4). A range of symptom frequencies and durations were reported. The most frequently reported symptoms were joint pain (n = 30; 100%), joint stiffness (n = 28; 93%) and swelling (n = 28; 93%). The joints most frequently reported as being painful were the knees (n = 19/29; 66%), hands (n = 17/29; 59%) and shoulders (n = 15/29; 52%). The vast majority of participants reported joint pain (n = 30/30; 100%), stiffness (n = 24/28; 86%) and swelling (n = 21/28; 75%) spontaneously, although fatigue was most frequently mentioned when probed (n = 14/21; 67%) (Fig. 4). Some participants reported that their joints became swollen approximately monthly (n = 4/22; 18%) or less often than monthly (n = 6/22; 27%), and almost half of participants reported that their swelling typically lasts a number of days (n = 10/21; 48%). Several less-common RA symptoms were also reported by a small number of participants, including: loss of appetite (n = 3; 10%), tingling (n = 3; 10%), joint locking (n = 2; 7%), general weakness (n = 2; 7%), carpal tunnel syndrome (n = 1; 3%), dry eyes (n = 1; 3%) and nausea (n = 1; 3%).

#### Impacts

**Fig. 5:**
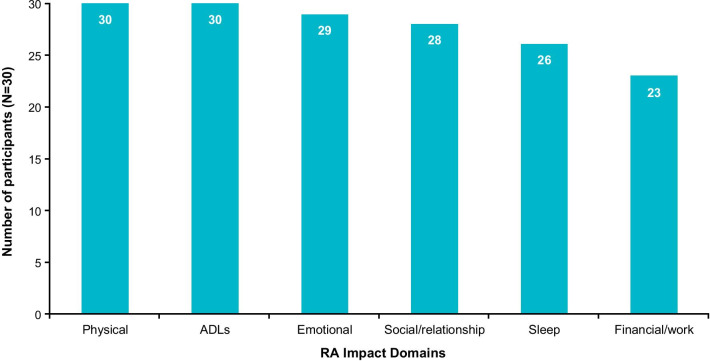
Impacts of RA reported by participants during concept elicitation. ADLs, activities of daily living; RA, rheumatoid arthritis

The impacts of RA on participants’ functioning and/or HRQoL, can be grouped into 6 domains: physical functioning, activities of daily living (ADLs), emotional wellbeing, social/relationships, sleep, and financial/work (Fig. 5). Physical functioning impacts were reported by all participants and included difficulties with: gripping (n = 28; 93%) and lifting objects (n = 25; 83%), mobility (n = 24; 80%), exercising (n = 22; 73%) and transitioning to standing (n = 21; 70%). Not all participants talked about how their symptoms impacted functioning; however, several participants noted that limitations on their physical functioning were primarily due to joint pain (n = 10/13; 85%) and stiffness (n = 7/13; 53%). Impacts on ADLs included difficulties with carrying out chores (n = 26; 87%), dressing (n = 22; 73%), preparing meals (n = 19; 63%), and self-care (n = 18; 60%) such as bathing/showering and brushing one’s hair. Almost all participants (n = 26; 87%) reported that their sleep was impacted by their RA symptoms (often related to sleeping position or simply disrupted sleep).

#### Conceptual model and supportive evidence for RASIQ

The findings of the concept elicitation interviews were used to develop an RA conceptual model (Additional file 1: Figure S1). Because the items in the RASIQ are meant to capture symptoms and impacts that may fluctuate daily, not all symptoms and impacts included in the RA conceptual model are addressed by items in the RASIQ v3.

## Discussion

According to previous research, rheumatologists value patient-reported measures of symptoms and physical function highly, as they provide important, complementary information to objective assessments, to guide treatment decisions [30]. Therefore, a PROM that is tailored to the RA population and provides comprehensive patient-reported insights into the symptoms and impacts of RA would be valuable to the field. This study developed and validated the RASIQ, a novel, robust RA-focused PROM. The RASIQ was developed in line with FDA guidance [11], using a mixed-method approach that considered both qualitative and quantitative data.

The final RASIQ instrument includes concepts identified by patients as being most important to their RA experience, and has reliable psychometric properties. The most important symptoms identified were pain (particularly joint pain), joint stiffness, and fatigue. Interestingly, pain and fatigue were reported to vary frequently, sometimes over the course of a day. The recall period for the RASIQ was set to ‘in the past 24 h'. After refinement in the first three rounds of cognitive debriefing interviews, the RASIQ included 16 items focused on joint pain and stiffness, energy/tiredness, and rest/sleep. The final set of cognitive debriefing interviews confirmed that participants found the items, instructions, and response options easy to understand, the items relevant, and the 24-h recall period appropriate.

Although not conducted as part of RASIQ development, an adjacent concept elicitation study identified concepts that are important to patients with RA and these were used to construct a conceptual model of RA symptoms and impacts, as opposed to forming items for inclusion in RASIQ. However, results from the concept elicitation study further validated the content of RASIQ, with joint pain, joint stiffness, and fatigue identified as core symptoms of RA. Concept elicitation also confirmed that sleep is regularly impacted by RA; this is consistent with previous studies which identified associations between RA-related pain and sleep disturbance [31–34], although the directionality of the association has not been fully elucidated [32, 35, 36]. The concept elicitation process identified more symptoms and impacts than those addressed in the RASIQ, including joint swelling (symptom) and domains of physical functioning, ADL, and emotional well-being (impacts). However, joint swelling was not added to the RASIQ because it was not identified as relevant during the first set of cognitive debriefing interviews and it was captured by items assessing joint pain. Physical functioning, ADL and emotional domains were not added to the RASIQ given the 24-h recall period; it was felt that these items would show change over a longer time period (days–weeks), and other PROMs such as HAQ-DI and Short-Form Health Survey already cater well to these domains [37, 38]. Previous studies suggest that patients do not oppose daily assessment of symptoms and impacts in the form of daily diaries when the data collected are relevant and important to their disease experiences [39]. The use of RASIQ in combination with PROMs that assess longer-term changes has the potential to facilitate a comprehensive holistic assessment of the RA patient experience.

The results of the psychometric evaluation further differentiate the RASIQ from existing RA-focused PROMs. While other instruments, such as the RAPID3, associate well with subjective measures but have only weak associations with objective measures [40], the RASIQ is able to differentiate patient groups when using both subjective (e.g. PtGA) measures of disease activity as well as those with objective components (e.g. DAS28). These results suggest that the RASIQ could be used to monitor disease activity in clinical practice. A 3-factor structure was chosen due to issues identified with a 5-factor structure; for example, the 5-factor Energy/Tiredness scale and the rest item had lower test–retest reliability than the other scales and items in the RASIQ. However, the 3-factor structure requires further clinical investigation.

Ultimately, the patient-centric approach, the use of psychometric evaluation, and the focus on items that are most important to patients with RA are key strengths of the RASIQ. Nevertheless, this study was not without limitations. Firstly, the structure and approach to PROM development used to develop the RASIQ may be considered unconventional. For example, concept elicitation interviews were conducted after the development of the initial RASIQ questionnaire, rather than before. Despite this, the results of the concept elicitation interviews support the concepts and items included in the RASIQ. Secondly, the version of the RASIQ used in the Phase II RENAISSANCE and BAROQUE trials, which provided the data for the psychometric evaluation, had not undergone the final set of cognitive debriefing interviews. However, early cognitive debriefing interviews allowed major problems to be addressed before the concept elicitation, and no changes were made to the RASIQ after the final set of cognitive debriefing interviews. The RASIQ was used in the Phase II studies at specific timepoints, rather than as a daily diary; therefore, while this format established the content validity, measurement properties, and reliability of the 24-h recall period as a first step in the development of the RASIQ, additional studies that use the RASIQ as a daily diary are required for further psychometric analysis, development of the scoring algorithm, and to assess its ability to detect change. Furthermore, participants were asked to self-report their RA diagnosis and disease severity in the final set of cognitive debriefing interviews, with no doctor’s confirmation required. To address this limitation, efforts were made to confirm each participant’s diagnosis using alternatives to medical notes; for example, patients were asked to bring their RA medication or prescription to the interview as evidence of their diagnosis. Finally, all interviews were conducted with English-speaking participants in the US; it is possible that other patient populations would report different RA experiences.

## Conclusions

The RASIQ is a novel, concise, 16-item, psychometrically-validated PROM that effectively captures the symptoms and impacts of RA that patients consider most important, that are not well covered by existing measures and that are susceptible to short-term fluctuations. Development of the RASIQ involved substantial patient input and psychometric evaluation, the results of which support the content and psychometric validity of the instrument. The RASIQ has the potential to be an important addition to the arsenal of RA-specific PROMs and complements existing RA disease activity measures. Future evaluations of the ability of RASIQ to measure daily fluctuations may allow a more granular assessment of key concepts, particularly those related to pain; if successful, the RASIQ could therefore be used alongside existing measures to better understand the unmet treatment needs of patients with RA.

## Supplementary Information

**Additional file 1**. Supplementary Data.

## Data Availability

Information on GlaxoSmithKline’s (GSK’s) data sharing commitments and requesting access to anonymized individual participant data and associated documents from GSK-sponsored studies can be found at: www.clinicalstudydatarequest.com.

## Abbreviations

ACR: American College of Rheumatology
ADL: Activities of daily living
CFA: Confirmatory factor analyses
CFI: Comparative fit index
CRP: C-reactive protein
csDMARD: Conventional synthetic DMARD
DAS: Disease activity score
DAS28: Disease Activity Score 28 joint count
D_LCO_: Diffusing capacity for carbon monoxide
DMARD: Disease-modifying antirheumatic drug
EFA: Exploratory factor analyses
EMA: European Medicines Agency
EULAR: European League Against Rheumatism
FDA: Food and Drug Administration
FEV_1_: Forced expiratory volume in 1 s
GED: General education diploma
HAQ-DI: Health Assessment Questionnaire-Disability Index
HRQoL: Health-related quality of life
ICC: Intraclass correlation coefficient
IRB: Institutional Review Board
MTX: Methotrexate
NSAID: Non-steroidal anti-inflammatory
NRS: Numerical rating scale
PhGA: Physician’s global assessment of disease activity
PtGA: Patient’s global assessment of disease activity
PRO: Patient-reported outcome
PROM: Patient-reported outcome measure
RA: Rheumatoid arthritis
RAID: Rheumatoid Arthritis Impact of Disease
RAPID: Routine Assessment of Patient Index Data
RASIQ: Rheumatoid Arthritis Symptom and Impact Questionnaire
RMSEA: Root mean square error of approximation
SD: Standard deviation
TLI: Tucker–Lewis index
tsDMARD: Targeted synthetic DMARD
VAS: Visual analog scale
