# Supplementary material for: Development, psychometric evaluation and cognitive debriefing of the rheumatoid arthritis symptom and impact questionnaire (RASIQ)
Source: J Patient Rep Outcomes. 2021 Dec 11;5:129. doi: 10.1186/s41687-021-00400-3 (PMC8665953; doi:10.1186/s41687-021-00400-3)
Supplement: Supplementary file 1 — Additional file 1. Supplementary Data. [file 41687_2021_400_MOESM1_ESM.docx]

# SUPPLEMENTARY DATA

## Supplementary Methods

### Qualitative search strategy

Literature searches were conducted individually in EMBASE, MEDLINE and PsycINFO by applying controlled vocabulary terms specific to each database. All searches were conducted on 2 October 2013.

**Database: Ovid EMBASE 2001 to 2013 Week 39. The number of search results is shown in brackets.**

- 1. qualitative research/ (22308)

1. focus group$.tw. (24418)
2. nursing methodology research/ (13706)
3. (patient$ adj4 (interview$ or perspective$ or journey$ or view$ or experience$)).tw. (155933)
4. nurs$ method$ research.tw. (0)
5. interview/ (118638)
6. phenomenology/ (6350)
7. grounded theory/ (1553)
8. content analysis/ (3462)
9. thematic analysis/ (2157)
10. 11 ((illness or disease) adj3 journey$).tw. (88)
11. (interview adj10 (ill$ or disease$ or patient$ or health$ or clinic$ or medic$)).tw. (40133)
12. semi structured interview/ (10017)
13. (Qualitative adj2 (research or interview$ or method$ or analys$)).tw. (35453)
14. qualitative analysis/ (31257)
15. Purposive sampl$.tw. (3291)
16. fatigue/ or exhaustion/ or lassitude/ (104935)
17. stiffness.mp. or rigidity/ (57428)
18. inflammation/ or chronic inflammation/ or inflammatory disease/ or musculoskeletal system inflammation/ or neurogenic inflammation/ or soft tissue inflammation/ (260190)
19. inflammation.mp. (216)
20. pain.mp. or pain/ (730263)
21. pain/ or bone pain/ or chronic pain/ or inflammatory pain/ or musculoskeletal pain/ or myalgia/ (249357)
22. rheumatoid arthritis/ or rheumatoid arthr*.mp. or rheumatology/ (166328)
23. 1 or 2 or 3 or 4 or 5 or 6 or 7 or 8 or 9 or 10 or 11 or 12 or 13 or 14 or 15 or 16 (375670)
24. tiredness.mp. (3813)
25. sleep/ (55593)
26. 17 or 25 or 26 (159013)
27. 21 or 22 (748148)
28. 18 or 19 or 20 or 27 or 28 (1157602)
29. 23 and 24 and 29 (1298)
30. limit 30 to (yr="2001 - 2014" and (adult <18 to 64 years> or aged <65+ years>)) (436)

**Database: Ovid MEDLINE(R) <1946 to September Week 3 2013>**

1. qualitative research/ (19324)
2. focus groups/ (17086)
3. nursing methodology research/ (15515)
4. (patient$ adj4 (interview$ or journey$ or experience$ or view$ or perspective$)).tw. (113147)
5. purposive sampl$.tw. (2586)
6. interviews as topic/ (43259)
7. ((illness or disease$) adj3 journey).tw. (52)
8. (interview$ adj10 (patient$ or health$ or clinic$ or medic$ or disease$ or ill$)).tw. (73284)
9. phenomenology.tw. (4520)
10. grounded theory.tw. (5291)
11. (qualitative adj2 (stud$ or research or interview$ or method$ or analys$)).tw. (42077)
12. semi$ structure$ interview$.tw. (10725)
13. 1 or 2 or 3 or 4 or 5 or 6 or 7 or 8 or 9 or 10 or 11 or 12 (261835)
14. fatigue/ (19447)
15. sleep/ (38495)
16. stiffness.mp. (34036)
17. inflammation/ (100342)
18. pain/ (110725)
19. 14 or 15 or 16 or 17 or 18 (296597)
20. arthritis, rheumatoid/ (83152)
21. 13 and 19 and 20 (190)
22. limit 21 to (yr="2001 -Current" and ("young adult (19 to 24 years)" or "adult (19 to 44 years)" or "young adult and adult (19-24 and 19-44)" or "middle age (45 to 64 years)" or "middle aged (45 plus years)" or "all aged (65 and over)" or "aged (80 and over)")) (92)

**Database: PsycINFO <1987 to September Week 4 2013>**

1. qualitative research/ (3798)
2. group discussion/ (1845)
3. focus group$.tw. (17940)
4. (patient$ adj4 (interview$ or journey$ or view$ or experience$ or perspective$)).tw. (23134)
5. purposive sampl$.tw. (2045)
6. interviews/ (4606)
7. (interview adj10 (patient$ or health$ or clinic$ or medic$ or disease$ or ill$ or physician$)).tw. (17515)
8. phenomenology/ (7440)
9. life experiences/ (14578)
10. grounded theory/ (1809)
11. content analysis/ (3194)
12. client attitudes/ (11392)
13. ((ill$ or disease$) adj2 journey$).tw. (53)
14. (qualitative adj2 (stud$ or research or method$ analys$ or interview$)).tw. (36110)
15. semi?structure$ interview$.tw. (5300)
16. (qualitative adj (stud$ or research or method$ or analys$ or interview$)).tw. (41412)
17. rheumatoid arthritis.mp. or exp Rheumatoid Arthritis/ (1865)
18. 1 or 2 or 3 or 4 or 5 or 6 or 7 or 8 or 9 or 10 or 11 or 12 or 13 or 14 or 15 or 16 (135802)
19. exp fatigue/ (4670)
20. exp sleep/ (12733)
21. stiffness.mp. (936)
22. exp inflammation/ (5342)
23. exp pain/ (35669)
24. 19 or 20 or 21 or 22 or 23 (57616)
25. 17 and 18 and 24 (39)
26. limit 25 to (("300 adulthood <age 18 yrs and older>" or 320 young adulthood <age 18 to 29 yrs> or 340 thirties <age 30 to 39 yrs> or 360 middle age <age 40 to 64 yrs> or "380 aged <age 65 yrs and older>" or "390 very old <age 85 yrs and older>") and yr="2014") (0)

### PRO instrument search strategy

Literature searches were conducted individually in EMBASE and MEDLINE by applying controlled vocabulary terms specific to each database. Both searches were run on 2 October 2013.

**Database: Embase 2001 to 2013 Week 39. The number of search results is shown in brackets.**

1. self-report/ (58418)
2. measurement/ (79266)
3. questionnaire/ (372343)
4. instrument/ (6795)
5. instrument validation/ (1613)
6. psychometry/ (42806)
7. fatigue/ or exhaustion/ or lassitude/ (104935)
8. inflammation/ or chronic inflammation/ or inflammatory disease/ or musculoskeletal system inflammation/ or neurogenic inflammation/ or soft tissue inflammation/ (260190)
9. pain/ or bone pain/ or chronic pain/ or inflammatory pain/ or musculoskeletal pain/ or myalgia/ (249357)
10. rheumatoid arthritis/ or rheumatoid arthr*.mp. or rheumatology.mp. [mp=title, abstract, subject headings, heading word, drug trade name, original title, device manufacturer, drug manufacturer, device trade name, keyword] (181623)
11. 1 or 2 or 3 or 4 or 5 or 6 (526884)
12. rigidity/ (17481)
13. sleep/ (55593)
14. 7 or 8 or 12 or 13 (431986)
15. 10 and 11 and 14 (1151)
16. limit 15 to (yr="2001 - 2014" and (adult <18 to 64 years> or aged <65+ years>)) (425)

**Database: Ovid MEDLINE(R) <1946 to September Week 3 2013>**

1. questionnaire/ (303293)
2. ((patient$ or symptom$) adj5 questionnaire$).tw. (34631)
3. ((patient$ or symptom$) adj5 measurement$).tw. (27238)
4. questionnaires/ (303293)
5. self-assessment/ (10589)
6. self-report/ (7463)
7. psychometrics/ (55463)
8. "Weights and Measures"/ (2377)
9. rheumatoid arthritis.mp. or exp Arthritis, Rheumatoid/ (117741)
10. 1 or 2 or 3 or 4 or 5 or 6 or 7 or 8 (397727)
11. fatigue/ (19447)
12. sleep/ (38495)
13. stiffness.mp. (34036)
14. inflammation/ or neurogenic inflammation/ (100833)
15. pain/ (110725)
16. 11 or 13 or 14 or 15 (259857)
17. 9 and 10 and 16 (607)
18. limit 17 to (yr="2001 -Current" and ("young adult (19 to 24 years)" or "adult (19 to 44 years)" or "young adult and adult (19-24 and 19-44)" or "middle age (45 to 64 years)" or "middle aged (45 plus years)" or "all aged (65 and over)" or "aged (80 and over)")) (333)

## Supplementary Figure 1: Conceptual model of RA symptoms and impacts, derived from the concept elicitation interviews


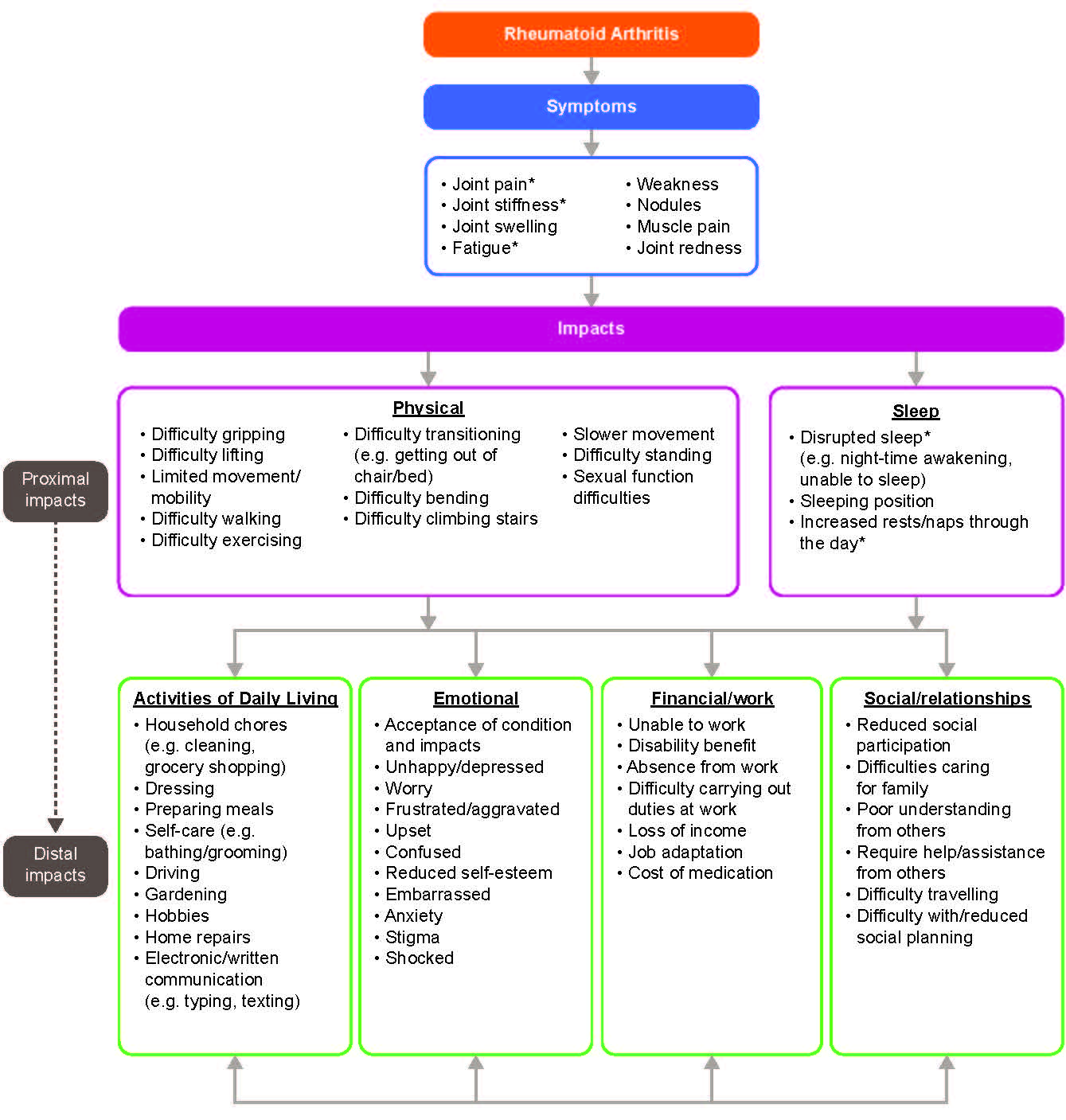


*Symptoms/impacts that were included in RASIQ

## Supplementary Table 1: A summary of the patient populations used in this study

| **Study part** | **Participants** | **Key inclusion criteria** | **Key exclusion criteria** |
| --- | --- | --- | --- |
| **Initial cognitive debriefing interviews** | - N=15 | - ≥18 years of age - Diagnosis of RA - Currently taking MTX at stable dose - Able to read and speak English at an adequate level to compete study tasks - In the opinion of the clinical investigator have moderately to severely active RA severity | - History of inflammatory rheumatological disorders other than RA - RA functional class IV of the ACR 1991 Revised Criteria for Global Functional Status in RA [1] (“Limited in ability to perform usual self-care, vocational, and avocational activities”) - Significant concurrent, uncontrolled medical condition including, but not limited to, renal, hepatic, haematological, gastrointestinal, endocrine, pulmonary, neurological, cerebral psychiatric disease, or active infections - Patients with newly diagnosed RA (<1 year since RA diagnosis) |
| **Concept elicitation interviews** | - N=30 | - ≥18 years of age - A diagnosis of RA and had this diagnosis for at least 6 months - Minimum of 4 swollen and tender joints - Either: - An inadequate responder to csDMARDs and has never received a bDMARD/tsDMARD or - An inadequate responder to bDMARDs/tsDMARDs (patient may be receiving bDMARDs/tsDMARDs only OR may be continuing csDMARD treatment in addition to receiving a bDMARD/tsDMARD) - Willing and able to participate in the study and provide written informed consent - Fluent US-English speaker and able to read, write and fully understand the US-English language - Willing and able to participate in a 60-minute interview to discuss their experience of RA | - A history of other inflammatory rheumatological or autoimmune disorders that are not secondary to RA - Significant unstable or uncontrolled acute or chronic disease other than RA - Unwilling or unable to comply with the requirements of the study or has a physical or mental condition that, in the opinion of the physician, may affect the participant’s ability to participate in the study, the responses he/she might provide or their ability to provide consent - Currently or previously (in the past year) enrolled in a clinical trial for RA |
| **Psychometric evaluation** | - BAROQUE N=205 - RENAISSANCE N=39 - Total N=244 | BAROQUE (GSK study 201755):   - ≥18 years of age - Clinical diagnosis of RA according to ACR/EULAR 2010 classification criteria - Functional class I, II, or III (1992 ACR Classification of Functional Status in RA) - Have at least 4 swollen and 4 tender joints - DAS28 CRP ≥3.2 at Screening, DAS28 erythrocyte sedimentation rate (ESR) ≥3.2 at Day 1, and CRP ≥5.0 mg/L - Previously received MTX for at least 12 weeks before screening (alongside oral folic acid) - D_LCO_ ≥60% and FEV_1_ ≥70% predicted   RENAISSANCE (GSK study 205180):   - ≥18 years of age - Clinical diagnosis of RA according to ACR/EULAR 2010 classification criteria - Functional class I, II, or III (1992 ACR Classification of Functional Status in RA) - Have at least 4 swollen and 4 tender joints - DAS28 CRP ≥3.2 at Screening, and CRP ≥5.0 mg/L - Presence of inflammation - Previously received MTX for at least 12 weeks before screening (alongside oral folic acid) | BAROQUE (GSK study 201755) and RENAISSANCE (GSK study 205180):   - History of other inflammatory, rheumatological or autoimmune disorders - History of any respiratory disease which may compromise safety or completion of participation - Persistent cough or unexplained dyspnea - Current or history of renal disease - Use of prohibited medication, including any conventional DMARD (other than MTX), biologic agents, alkylating agents, corticosteroids or NSAIDS |
| **Final cognitive debriefing interviews** | - N=12 | - ≥18 years of age - Self-reported clinician diagnosis of moderate to severe RA - Received the diagnosis at ≥18 years of age, ≥2 years ago, and received treatment for RA in the past  2 years - Experienced symptoms of RA (e.g., joint pain/swelling) in the past 7 days - Received a DMARD and/or a biologic treatment - Have ≥6 swollen joints and ≥6 tender joints - Able to read, write, speak, and comprehend English fluently - Able to provide written informed consent | - Unwilling or unable to participate in a 60-minute interview |

ACR, American College of Rheumatology; bDMARD, biological DMARD; CRP, C-reactive protein; csDMARD, conventional synthetic DMARD; DAS28, Disease Activity Score 28 joint count; D_LCO_; diffusing capacity for carbon monoxide; DMARD, disease-modifying antirheumatic drug; EULAR, European League Against Rheumatism; FEV_1_; forced expiratory volume in 1 second; MTX, methotrexate; NSAID, nonsteroidal anti-inflammatory drug; RA, rheumatoid arthritis; tsDMARD, targeted synthetic DMARD.

## Supplementary Table 2: Overview of RASIQ item content

| **Item Label** | **Item Content** | **Range of Item Response Values** |
| --- | --- | --- |
| JP01 | In the past 24 hours, how severe was your joint pain at its worst when sitting? | 0-10 |
| JP02 | In the past 24 hours, how severe was your joint pain at its worst when lying down? | 0-10 |
| JP03 | In the past 24 hours, how severe was your joint pain at its worst when you started to move after being still? | 0-10 |
| JP04 | In the past 24 hours, how severe was your joint pain at its worst when walking? | 0-10 |
| JP05 | In the past 24 hours, how severe was your joint pain at its worst when in a standing position? | 0-10 |
| JP06 | In the past 24 hours, how severe was your joint pain at its worst when lifting objects? | 0-10 |
| JP07 | In the past 24 hours, how severe was your joint pain at its worst? | 0-10 |
| JS01 | In the past 24 hours, how often have you had joint stiffness? | 1-5 |
| JS02 | When you woke up today, how severe was the early morning stiffness in your joints? | 1-5 |
| JS03 | How long did the early morning stiffness in your joints last today? | 1-6 |
| JS04 | In the past 24 hours, how severe was your joint stiffness at its worst later in the day? | 1-5 |
| ET01 | In the past 24 hours, how much energy did you have? | 1-5 |
| ET02 | In the past 24 hours, how often have you felt physically tired? | 1-5 |
| RE01 | In the past 24 hours, how often have you had to rest because of your joint pain? | 1-5 |
| SL01 | Last night, how was the quality of your sleep? | 1-5 |
| SL02 | Last night, how much was your sleep affected by joint pain? | 1-5 |

## Scoring algorithm for RASIQ

A scoring algorithm based on summation of items within each scale followed by a transformation to a 0–100 scale is proposed for scoring the RASIQ. All response scales are assumed to be reported using discrete continuous scales as shown in Supplementary Table 9. All items and scales are scored so that a higher score indicates greater impairment. All items should be checked for out-of-range response values before recoding. Currently, no methods have been proposed to estimate the missing item responses. The process for obtaining the 0–100 scores for the RASIQ scales is as follows:

Step 1: Recode item response values

Item response values are recoded for 2 items (ET01: In the past 24 hours, how much energy did you have? and SL01: Last night, how was the quality of your sleep?). Specifically, the item response values for these 2 items are reverse-coded to ensure that higher item scores indicate greater impairment.

Step 2: Determine scale total raw scores

After item recoding, a total raw score is computed for each scale. The total raw score is the simple algebraic sum of the final response values for all the items in a given scale. Results from Mokken scale analysis (MSA)15, conducted to evaluate whether the RASIQ response options are correctly ordered, supported the monotonicity property of the RASIQ response options and appropriateness of employing the summated scoring algorithm.

Step 3: Transform scale total raw scores to 0–100 scale scores

After each scale total raw score is derived, a 0–100 score for each scale of the RASIQ can be derived using the following formula:

$$\frac{(Actual scale total raw score-Lowest possible scale total raw score )}{Possible range of scale total raw score (\max-min)}$$

## Supplementary Table 3: Articles identified describing focus groups or interviews with patients with rheumatoid arthritis

| **Reference** | **Concepts identified** | **Summary of study** |
| --- | --- | --- |
| Ahlmén M, Nordenskiöld U, Archenholtz B, et al. Rheumatology outcomes: the patient's perspective. A multicentre focus group interview study of Swedish rheumatoid arthritis patients. Rheumatology (Oxford, England). 2005;44(1):105-110. | Pain, Fatigue, Stiffness,  Satisfaction | Study to understand how patients assess outcomes, in order to update current measures.  Involved focus groups of patients with RA.  N=25 |
| Ahlstrand I, Björk M, Thyberg I, Börsbo B, Falkmer T. Pain and daily activities in rheumatoid arthritis. Disability and rehabilitation. 2012;34(15):1245-1253. | Pain | Study to describe RA patients experiences of pain and relationship to daily activities.  Involved semi-structured focus groups of patients with RA.  N=33 |
| Al Attia HM, Al Abbasi M. Sensing the main health concerns in patients with established rheumatoid arthritis. Clinical Rheumatology. 2011;30(11):1511. | Pain, Fatigue, Stiffness | Study to determine the spectrum and prioritisation of health concerns likely to be present in patients with RA.  Involved interviews and questionnaires with patients with RA and arthropathies.  N=101 |
| Bartlett SJ, Hewlett S, Bingham CO, 3rd, et al. Identifying core domains to assess flare in rheumatoid arthritis: an OMERACT international patient and provider combined Delphi consensus. Annals of the rheumatic diseases. 2012;71(11):1855-1860. | Pain, Fatigue, Joint Swelling, Tenderness, Stiffness | Study to define RA flares from patients' and health care professionals' perspectives with the aim of developing a measure for RA flare.  Involved a Delphi study with patients with RA and health care professionals.  N=125 |
| Carr A, Hewlett S, Hughes R, et al. Rheumatology outcomes: the patient's perspective. The Journal of rheumatology. 2003;30(4):880-883. | Pain, Fatigue, Wellbeing, Mobility | Study to determine which outcomes are important from the patients' perspective. e.g. physical (pain, disability, deformity), general health (fatigue, feeling well), emotional impact.  Involved focus groups of patients with RA.  N=29 |
| Conner TS, Tennen H, Zautra AJ, Affleck G, Armeli S, Fifield J. Coping with rheumatoid arthritis pain in daily life: within-person analyses reveal hidden vulnerability for the formerly depressed. Pain. 2006;126(1-3):198-209. | Pain, Depression | Study To examine the association between history of depression and day-to-day coping with RA pain.  Involved end-of-day ratings/diary completed by patients with RA, prior to sleep, for 30 days.  N=188 |
| Lempp H, Hofmann D, Hatch SL, Scott DL. Patients' views about treatment with combination therapy for rheumatoid arthritis: a comparative qualitative study. BMC musculoskeletal disorders. 2012;13:200. | Expectations about combined therapy; impact on quality of life; concerns about treatment; home management of treatment | Study to explore the expectations regarding the impact on quality of life, concerns and management of patients with newly diagnosed and established RA when in receipt of combination therapy.  Involved interviews with outpatients with RA  N=18 |
| Nikolaus S, Bode C, Taal E, van de Laar MA. Four different patterns of fatigue in rheumatoid arthritis patients: results of a Q-sort study. Rheumatology (Oxford, England). 2010;49(11):2191-2199. | Fatigue | Study to gain insight into the experience of fatigue in RA.  Involved in-depth structured interviews and VAS scoring of patients with RA.  N=31 |
| Nikolaus S, Bode C, Taal E, van der Laar MA. Expert evaluations of fatigue questionnaires used in rheumatoid arthritis: a Delphi study among patients, nurses and rheumatologists in the Netherlands. Clinical and experimental rheumatology. 2012;30(1):79-84. | Fatigue | Study to determine whether an item pool for a PRO for fatigue contained all relevant aspects to assess fatigue in RA.  Involved a Delphi study with rheumatologists, nurses and patients with RA.  N=111 |
| Nikolaus S, Bode C, Taal E, vd Laar MA. Selection of items for a computer-adaptive test to measure fatigue in patients with rheumatoid arthritis: a Delphi approach. Quality of life research : an international journal of quality of life aspects of treatment, care and rehabilitation. 2012;21(5):863-872. | Fatigue | Study to explore and select the relevant dimensions of fatigue for the development of a measure to assess fatigue in patients with RA.  Involved a Delphi study with rheumatologists, nurses, and patients.  N=111 |
| Repping-Wuts H, Uitterhoeve R, van Riel P, van Achterberg T. Fatigue as experienced by patients with rheumatoid arthritis (RA): a qualitative study. International journal of nursing studies. 2008;45(7):995-1002. | Fatigue | Study to explore the experience of fatigue in RA from the patient perspective.  Involved interviews with patients with RA.  N=29 |
| Sanderson T, Morris M, Calnan M, Richards P, Hewlett S. What outcomes from pharmacologic treatments are important to people with rheumatoid arthritis? Creating the basis of a patient core set. Arthritis care & research. 2010;62(5):640-646. | Pain, Fatigue, Quality of life | Study to elicit patient priority treatment outcomes for pharmacologic interventions with a focus on fatigue and quality of life.  Involved in-depth interviews with patients with RA.  N=23 |
| Sanderson T, Morris M, Calnan M, Richards P, Hewlett S. Patient perspective of measuring treatment efficacy: the rheumatoid arthritis patient priorities for pharmacologic interventions outcomes. Arthritis care & research. 2010;62(5):647-656. | Fatigue, Coping, Life enjoyment | Study to develop a complementary core set of concepts using patient input to promote the inclusion of their priority outcomes for pharmacologic interventions.  Involved interviews, surveys, nominal groups, ranking and discussion with patients with RA.  N=26 |
| Tuominen R, Tuominen S, Möttönen T. Consistency of assessments and willingness to pay for a reduction in morning symptoms over time in patients with rheumatoid arthritis. Scandinavian journal of rheumatology. 2012;41(6):438-441. | Pain, Fatigue, Stiffness, Morning Stiffness | Study to determine the variation in morning symptoms and in the corresponding amounts patients would be willing to pay to reduce them.  Involved interviews with patients with RA and a numeric rating scale.  N=100 |
| Tuominen R, Azbel M, Hemmilä J, Möttönen T. Willingness to pay for improvement of physical function among rheumatoid arthritis patients as measured by Health Assessment Questionnaire. Rheumatology international. 2011;31(3):347-352. | Pain | Study to assess willingness to pay to reduce pain.  Involved health assessment questionnaire and VAS in patients with RA.  N=242 |
| Tuominen R, Tuominen S, Möttönen T. How much is a reduction in morning stiffness worth to patients with rheumatoid arthritis? Scandinavian Journal of Rheumatology. 2011;40(sup125):12-16. | Stiffness | Study to determine the monetary equivalent of the emotional and functional impact of morning stiffness in RA patients.  Involved interviews with patients with RA, human capital approach, marginal value of time, and willingness to pay.  N=166 |
| van Hoogmoed D, Fransen J, Bleijenberg G, van Riel P. Physical and psychosocial correlates of severe fatigue in rheumatoid arthritis. Rheumatology (Oxford, England). 2010;49(7):1294-1302. | Pain, Fatigue | Study to determine the prevalence of severe fatigue in RA, patient perceptions of fatigue, disease-related and psychosocial factors associated with fatigue severity.  Involved clinical data, and questionnaire completed by patients with RA.  N=228 |

## Supplementary Table 4: Participant demographics and characteristics for the initial cognitive debriefing interviews

| **Characteristic** | **Initial cognitive debriefing interviews (N=15)** |
| --- | --- |
| Age, years, mean (SD) | 57 (9) |
| Female, n (%) | 13 (87) |
| Ethnicity, n (%) |  |
| White/Caucasian | 13 (87) |
| Black/African American | 2 (13) |
| Time since RA diagnosis, years, mean (SD) | 12.3 (7) |
| Education, n (%) |  |
| High school diploma or equivalent | 6 (40) |
| Bachelor’s degree | 4 (27) |
| Graduate degree | 2 (13) |
| Associate degree | 2 (13) |
| Did not complete high school | 1 (7) |
| Employment, n (%) |  |
| Full time | 6 (40) |
| Disabled | 4 (27) |
| Retired | 2 (13) |
| Homemaker | 1 (7) |
| Unemployed | 1 (7) |
| Other | 1 (7) |
| Living arrangement, n (%) |  |
| With spouse/partner | 9 (60) |
| With other family members | 2 (13) |
| Alone | 3 (20) |
| Other | 1 (7) |
| Number of flares in previous month, n (%) |  |
| 0 | 2 (13) |
| 1 | 4 (27) |
| 2 | 0 (0) |
| 3 | 1 (7) |
| 4+ | 8 (53) |
| Currently experiencing a flare, n (%) | 9 (60) |
| Suffers from morning stiffness, n (%) | 13 (87) |
| Duration of morning stiffness, minutes, mean (SD) | 85 (81) |
| Concomitant medications, n (%) |  |
| Methotrexate | 15 (100) |
| NSAIDs | 3 (20) |
| Other^a^ | 3 (20) |
| None | 8 (53) |
| Comorbidities |  |
| Hypertension | 5 (33) |
| Diabetes | 1 (7) |
| Depression | 1 (7) |
| Osteoporosis | 1 (7) |
| Hypercholesterolaemia | 1 (7) |
| Hyperthyroid | 1 (7) |
| None | 6 (40) |

^a^Includes multivitamin, insulin, antibiotics, and other pain relief

NSAID, non-steroidal anti-inflammatory; RA, rheumatoid arthritis; SD, standard deviation

## Supplementary Table 5: Conceptual framework for RASIQ v3

| **Scale** | **Item** |
| --- | --- |
| **Symptoms** | |
| Joint Pain | Severity of joint pain when sitting (past 24 hours) |
|  | Severity of joint pain when lying down (past 24 hours) |
|  | Severity of joint pain when starting to move after being still (past 24 hours) |
|  | Severity of joint pain when walking (past 24 hours) |
|  | Severity of joint pain when standing (past 24 hours) |
|  | Severity of joint pain when lifting objects (past 24 hours) |
|  | Severity of joint pain at its worst (past 24 hours) |
| Energy/tiredness | Level of energy (past 24 hours) |
|  | Frequency of physical tiredness (past 24 hours) |
| Joint stiffness | Frequency of joint stiffness (past 24 hours) |
|  | Severity of early morning stiffness in joints (when woke up today) |
|  | Duration of early morning stiffness in joints (today) |
|  | Severity of joint stiffness in the day (past 4 hours) |
| **Impacts** | |
| Rest | Frequency of rest due to joint pain (past 24 hours) |
| Sleep | Sleep quality (last night) |
|  | Sleep disturbance due to joint pain (last night) |

## Supplementary Table 6: Participant demographics and characteristics for the final cognitive debriefing interviews

| **Characteristic** | **Final cognitive debriefing interviews (N=12)** |
| --- | --- |
| Age, years, mean (SD) | 53 (16) |
| Range | 23–68 |
| Time since RA diagnosis, years, mean (SD) | 13 (12) |
| Range | 2–30 |
| Age at RA diagnosis, years, n (%) |  |
| ≥25 | 4 (33) |
| 26–35 | 0 (0) |
| 36–45 | 4 (33) |
| 46–55 | 3 (25) |
| ≥56 | 1 (8) |
| Female, n (%) | 8 (67) |
| Race/ethnicity, n (%) |  |
| White/Caucasian | 7 (58) |
| African American | 2 (17) |
| Asian/East Asian | 1 (8) |
| Mixed Race | 1 (8) |
| Other | 1 (8) |
| Self-reported severity of RA, n (%) |  |
| Moderate | 9 (75) |
| Severe | 3 (25) |
| Self-reported RA stage, n (%) |  |
| Stage I  Stage II  Stage III  Stage IV  Does not know/not told by doctor  Medication currently taking for RA, n (%)  Biologic Only  DMARD Only  Both Biologic and DMARD  Neither Biologic or DMARD | 1 (8)  0 (0)  0 (0)  2 (17)  9 (75)  1 (8)  3 (25)  8 (67)  0 (0) |

DMARD, disease-modifying anti-rheumatic drug; RA, rheumatoid arthritis; SD, standard deviation.

## Supplementary Table 7: Known-group analyses assessing validity of RASIQ at Week 12 versus disease activity measures (5-factor structure: Joint Pain, Energy/Tiredness, Joint Stiffness, Rest and Sleep; 3-factor structure: Joint Pain, Joint Stiffness and Impact)

| **DAS28(ESR)** | | | | | | | | | | | | | | | | | | | | | | | | | | | | | | | | | | | | | | | | | | | | | | | | |
| --- | --- | --- | --- | --- | --- | --- | --- | --- | --- | --- | --- | --- | --- | --- | --- | --- | --- | --- | --- | --- | --- | --- | --- | --- | --- | --- | --- | --- | --- | --- | --- | --- | --- | --- | --- | --- | --- | --- | --- | --- | --- | --- | --- | --- | --- | --- | --- | --- |
|  | | | **1. High disease activity** | | | | | | | | | | | | **2. Moderate disease activity** | | | | | | | | | | | | **3. Low disease activity/disease remission** | | | | | | | | | | | | **Bonferroni-adjusted P** | | | | | | |  | | |
| **Scale** | | | **N** | | | | **Mean** | | | | **SD** | | | | **N** | | | | **Mean** | | | | **SD** | | | | **N** | | | | **Mean** | | | | **SD** | | | | **Group 1 vs 2** | | | | **Group 2 vs 3** | | | **F** | | **P** |
| Joint Pain | | | 134 | | | | 60.43 | | | | 20.34 | | | | 66 | | | | 40.28 | | | | 19.59 | | | | 28 | | | | 24.18 | | | | 11.89 | | | | <0.0001 | | | | 0.0010 | | | 51.83 | | <0.0001 |
| Energy/Tiredness | | | 134 | | | | 59.30 | | | | 19.01 | | | | 66 | | | | 48.67 | | | | 16.86 | | | | 28 | | | | 35.19 | | | | 17.35 | | | | 0.0004 | | | | 0.0041 | | | 22.70 | | <0.0001 |
| Joint Stiffness | | | 134 | | | | 51.04 | | | | 18.14 | | | | 66 | | | | 37.43 | | | | 17.89 | | | | 28 | | | | 24.62 | | | | 14.23 | | | | <0.0001 | | | | 0.0051 | | | 31.41 | | <0.0001 |
| Rest | | | 134 | | | | 51.13 | | | | 21.07 | | | | 66 | | | | 35.61 | | | | 22.81 | | | | 28 | | | | 22.22 | | | | 17.45 | | | | <0.0001 | | | | 0.0187 | | | 26.73 | | <0.0001 |
| Sleep | | | 134 | | | | 49.91 | | | | 19.91 | | | | 66 | | | | 38.26 | | | | 19.72 | | | | 28 | | | | 28.24 | | | | 16.11 | | | | 0.0003 | | | | 0.0754 | | | 17.87 | | <0.0001 |
| Impact* | | | 134 | | | | 53.91 | | | | 17.10 | | | | 66 | | | | 41.89 | | | | 16.73 | | | | 28 | | | | 29.81 | | | | 13.48 | | | | <0.0001 | | | | 0.0050 | | | 28.96 | | <0.0001 |
| **DAS28(CRP)** | | | | | | | | | | | | | | | | | | | | | | | | | | | | | | | | | | | | | | | | | | | | | | | | |
|  | **1. High disease activity** | | | | | | | | | | | **2. Moderate disease activity** | | | | | | | | | | | | | | **3. Low disease activity/disease remission** | | | | | | | | | | | | **Bonferroni-adjusted P** | | | | | | |  | | | |
| **Scale** | **N** | | | **Mean** | | | | **SD** | | | | **N** | | | | **Mean** | | | | | | **SD** | | | | **N** | | | | **Mean** | | | | **SD** | | | | **1 vs 2** | | | | **2 vs 3** | | | **F** | | | **P** |
| Joint Pain | 111 | | | 61.62 | | | | 21.03 | | | | 72 | | | | 46.82 | | | | | | 19.24 | | | | 42 | | | | 25.41 | | | | 11.50 | | | | <0.0001 | | | | <0.0001 | | | 56.65 | | | <0.0001 |
| Energy/  Tiredness | 111 | | | 60.57 | | | | 19.39 | | | | 72 | | | | 51.23 | | | | | | 17.31 | | | | 42 | | | | 37.50 | | | | 14.61 | | | | 0.0022 | | | | 0.0003 | | | 25.81 | | | <0.0001 |
| Joint Stiffness | 111 | | | 51.76 | | | | 18.95 | | | | 72 | | | | 41.84 | | | | | | 16.59 | | | | 42 | | | | 27.31 | | | | 16.98 | | | | 0.0010 | | | | 0.0001 | | | 29.22 | | | <0.0001 |
| Rest | 111 | | | 52.95 | | | | 21.35 | | | | 72 | | | | 38.73 | | | | | | 21.87 | | | | 42 | | | | 24.40 | | | | 17.88 | | | | <0.0001 | | | | 0.0016 | | | 30.53 | | | <0.0001 |
| Sleep | 111 | | | 51.82 | | | | 20.31 | | | | 72 | | | | 41.02 | | | | | | 19.27 | | | | 42 | | | | 28.87 | | | | 15.24 | | | | 0.0008 | | | | 0.0038 | | | 23.20 | | | <0.0001 |
| Impact* | 111 | | | 55.55 | | | | 17.60 | | | | 72 | | | | 44.65 | | | | | | 16.22 | | | | 42 | | | | 31.43 | | | | 12.16 | | | | <0.0001 | | | | 0.0001 | | | 35.02 | | | <0.0001 |
| **Patient’s Assessment of Arthritis Pain** | | | | | | | | | | | | | | | | | | | | | | | | | | | | | | | | | | | | | | | | | | | | | | | | |
|  | | **Severe** | | | | | | | | | | | **Moderate** | | | | | | | | | | | **None to mild** | | | | | | | | | | | | **Bonferroni-adjusted P** | | | | | | | |  | | | | |
| **Scale** | | **N** | | | **Mean** | | | | **SD** | | | | **N** | | | | **Mean** | | | **SD** | | | | **N** | | | | **Mean** | | | | **SD** | | | | **Group 1 vs 2** | | | | **Group 2 vs 3** | | | | **F** | | | **P** | |
| Joint Pain | | 52 | | | 76.33 | | | | 11.17 | | | | 95 | | | | 54.30 | | | 15.89 | | | | 82 | | | | 28.80 | | | | 15.26 | | | | <0.0001 | | | | <0.0001 | | | | 169.34 | | | <0.0001 | |
| Energy/Tiredness | | 52 | | | 69.12 | | | | 15.28 | | | | 95 | | | | 56.97 | | | 16.18 | | | | 82 | | | | 39.04 | | | | 16.70 | | | | <0.0001 | | | | <0.0001 | | | | 58.33 | | | <0.0001 | |
| Joint Stiffness | | 52 | | | 59.86 | | | | 15.77 | | | | 95 | | | | 46.93 | | | 17.33 | | | | 82 | | | | 30.21 | | | | 15.58 | | | | <0.0001 | | | | <0.0001 | | | | 54.13 | | | <0.0001 | |
| Rest | | 52 | | | 62.25 | | | | 17.59 | | | | 95 | | | | 47.11 | | | 18.90 | | | | 82 | | | | 26.23 | | | | 20.12 | | | | <0.0001 | | | | <0.0001 | | | | 59.53 | | | <0.0001 | |
| Sleep | | 52 | | | 60.05 | | | | 18.54 | | | | 95 | | | | 47.24 | | | 17.88 | | | | 82 | | | | 29.78 | | | | 15.88 | | | | <0.0001 | | | | <0.0001 | | | | 50.66 | | | <0.0001 | |
| Impact* | | 52 | | | 64.12 | | | | 13.55 | | | | 95 | | | | 51.11 | | | 14.37 | | | | 82 | | | | 32.78 | | | | 14.19 | | | | <0.0001 | | | | <0.0001 | | | | 82.38 | | | <0.0001 | |
| **Patient’s Global Assessment of Arthritis** | | | | | | | | | | | | | | | | | | | | | | | | | | | | | | | | | | | | | | | | | | | | | | | | |
|  | | **1. Severe** | | | | | | | | | | | **2. Moderate** | | | | | | | | | | | **3. None to mild** | | | | | | | | | | | | **Bonferroni-adjusted P** | | | | | | | |  | | | | |
| **Scale** | | **N** | | | **Mean** | | | | **SD** | | | | **N** | | | | **Mean** | | | **SD** | | | | **N** | | | | **Mean** | | | | **SD** | | | | **Group 1 vs 2** | | | | **Group 2 vs 3** | | | | **F** | | | **P** | |
| Joint Pain | | 56 | | | 74.70 | | | | 12.90 | | | | 97 | | | | 53.14 | | | 16.57 | | | | 76 | | | | 28.29 | | | | 15.12 | | | | <0.0001 | | | | <0.0001 | | | | 149.74 | | | <0.0001 | |
| Energy/Tiredness | | 56 | | | 68.41 | | | | 15.56 | | | | 97 | | | | 55.93 | | | 17.03 | | | | 76 | | | | 38.83 | | | | 16.13 | | | | <0.0001 | | | | <0.0001 | | | | 53.84 | | | <0.0001 | |
| Joint Stiffness | | 56 | | | 59.36 | | | | 17.36 | | | | 97 | | | | 45.48 | | | 16.71 | | | | 76 | | | | 30.43 | | | | 16.05 | | | | <0.0001 | | | | <0.0001 | | | | 48.63 | | | <0.0001 | |
| Rest | | 56 | | | 61.82 | | | | 17.25 | | | | 97 | | | | 45.62 | | | 20.09 | | | | 76 | | | | 26.00 | | | | 19.47 | | | | <0.0001 | | | | <0.0001 | | | | 56.51 | | | <0.0001 | |
| Sleep | | 56 | | | 58.64 | | | | 19.38 | | | | 97 | | | | 45.88 | | | 18.46 | | | | 76 | | | | 30.50 | | | | 16.08 | | | | 0.0001 | | | | <0.0001 | | | | 40.04 | | | <0.0001 | |
| Impact* | | 56 | | | 63.18 | | | | 14.41 | | | | 97 | | | | 49.85 | | | 14.80 | | | | 76 | | | | 32.93 | | | | 14.40 | | | | <0.0001 | | | | <0.0001 | | | | 70.53 | | | <0.0001 | |
| **Physician’s Global Assessment of Arthritis** | | | | | | | | | | | | | | | | | | | | | | | | | | | | | | | | | | | | | | | | | | | | | | | | |
|  | | **1. Severe** | | | | | | | | | | | | **2. Moderate** | | | | | | | | | | | **3. None to mild** | | | | | | | | | | | | **Bonferroni-adjusted P** | | | | | | | |  | | | |
| **Scale** | | **N** | | | | **Mean** | | | | **SD** | | | | **N** | | | | **Mean** | | | **SD** | | | | **N** | | | | **Mean** | | | | **SD** | | | | **1 vs 2** | | | | **2 vs 3** | | | | **F** | | **P** | |
| Joint Pain | | 41 | | | | 68.50 | | | | 21.00 | | | | 85 | | | | 57.21 | | | 17.53 | | | | 102 | | | | 37.49 | | | | 20.90 | | | | 0.0097 | | | | <0.0001 | | | | 43.64 | | <0.0001 | |
| Energy/Tiredness | | 41 | | | | 64.06 | | | | 21.96 | | | | 85 | | | | 57.59 | | | 15.40 | | | | 102 | | | | 45.71 | | | | 19.47 | | | | 0.2122 | | | | <0.0001 | | | | 17.50 | | <0.0001 | |
| Joint Stiffness | | 41 | | | | 59.41 | | | | 19.06 | | | | 85 | | | | 46.50 | | | 16.30 | | | | 102 | | | | 35.76 | | | | 18.82 | | | | 0.0007 | | | | 0.0002 | | | | 26.27 | | <0.0001 | |
| Rest | | 41 | | | | 58.75 | | | | 21.60 | | | | 85 | | | | 47.32 | | | 19.79 | | | | 102 | | | | 33.82 | | | | 22.70 | | | | 0.0182 | | | | <0.0001 | | | | 21.77 | | <0.0001 | |
| Sleep | | 41 | | | | 53.13 | | | | 22.95 | | | | 85 | | | | 47.92 | | | 17.87 | | | | 102 | | | | 37.25 | | | | 20.05 | | | | 0.5187 | | | | 0.0010 | | | | 11.78 | | <0.0001 | |
| Impact* | | 41 | | | | 58.63 | | | | 20.03 | | | | 85 | | | | 51.67 | | | 14.17 | | | | 102 | | | | 39.95 | | | | 17.88 | | | | 0.1032 | | | | <0.0001 | | | | 21.09 | | <0.0001 | |

*Impact scale is a component of the 3-factor structure and brings together items from the Energy/Tiredness (2 items), Rest (1 item) and Sleep (2 items) scales.

## Supplementary Table 8: Known-group analyses assessing responsiveness of RASIQ from baseline to Week 12 (5-factor structure: Joint Pain, Energy/Tiredness, Joint Stiffness, Rest and Sleep; 3-factor structure: Joint Pain, Joint Stiffness and Impact)

| **ACR Response Criteria** | | | | | | | | | | | |
| --- | --- | --- | --- | --- | --- | --- | --- | --- | --- | --- | --- |
|  | **1. ACR20**  **(N=140)** | | | **2. ACR20-<ACR50**  **(N=42)** | | **3. ACR50+**  **(N=44)** | | **Bonferroni-adjusted** | |  | |
| **Scale** | **Mean** | | **SD** | **Mean** | **SD** | **Mean** | **SD** | **1 vs 2** | **2 vs 3** | **F** | **P** |
| Joint Pain | -8.45 | | 17.84 | -22.55 | 14.91 | -41.30 | 16.40 | <0.0001 | <0.0001 | 64.01 | <0.0001 |
| Energy/  Tiredness | -4.84 | | 17.89 | -14.29 | 20.02 | -26.99 | 16.61 | 0.0101 | 0.0039 | 25.90 | <0.0001 |
| Joint  Stiffness | -10.69 | | 17.91 | -21.85 | 16.50 | -33.16 | 19.03 | 0.0015 | 0.0112 | 28.01 | <0.0001 |
| Rest | -6.02 | | 22.39 | -17.26 | 25.60 | -38.07 | 24.99 | 0.0219 | 0.0002 | 31.23 | <0.0001 |
| Sleep | -6.57 | | 19.18 | -16.07 | 18.37 | -25.85 | 17.35 | 0.0130 | 0.0481 | 18.87 | <0.0001 |
| Impact* | -5.77 | | 15.39 | -15.60 | 15.63 | -28.75 | 14.67 | 0.0010 | 0.0003 | 38.92 | <0.0001 |
| **EULAR Response Categories (DAS28[CRP])** | | | | | | | | | | | |
|  | | **1. No Response (N=105)** | | **2. Moderate Response (N=79)** | | **3. Good Response**  **(N= 40)** | | **Bonferroni-adjusted P** | |  | |
| **Scale** | | **Mean** | **SD** | **Mean** | **SD** | **Mean** | **SD** | **1 vs 2** | **2 vs 3** | **F** | **P** |
| Joint Pain | | -9.15 | 16.08 | -18.32 | 21.92 | -38.46 | 16.90 | 0.0033 | <0.0001 | 36.08 | <0.0001 |
| Energy/Tiredness | | -4.41 | 17.82 | -13.61 | 20.53 | -23.75 | 16.70 | 0.0035 | 0.0166 | 16.47 | <0.0001 |
| Joint Stiffness | | -10.03 | 18.39 | -19.73 | 17.77 | -30.59 | 20.60 | 0.0018 | 0.0088 | 18.68 | <0.0001 |
| Rest | | -3.92 | 23.27 | -18.35 | 23.92 | -34.38 | 26.97 | 0.0003 | 0.0023 | 24.23 | <0.0001 |
| Sleep | | -5.02 | 18.22 | -15.98 | 19.61 | -23.13 | 19.72 | 0.0005 | 0.1618 | 15.46 | <0.0001 |
| Impact* | | -4.56 | 15.56 | -15.51 | 16.14 | -25.63 | 16.26 | <0.0001 | 0.0036 | 27.75 | <0.0001 |

| **EULAR Response Categories (DAS28[ESR])** | | | | | | | | | | |
| --- | --- | --- | --- | --- | --- | --- | --- | --- | --- | --- |
|  | **1. No Response (N=105)** | | **2. Moderate Response (N=79)** | | **3. Good Response**  **(N= 40)** | | **Bonferroni-adjusted P** | |  | |
| **Scale** | **Mean** | **SD** | **Mean** | **SD** | **Mean** | **SD** | **1 vs 2** | **2 vs 3** | **F** | **P** |
| Joint Pain | -9.96 | 17.15 | -20.25 | 22.34 | -38.25 | 15.80 | 0.0007 | <0.0001 | 24.47 | <0.0001 |
| Energy/Tiredness | -4.17 | 18.56 | -15.73 | 20.01 | -22.69 | 15.52 | <0.0001 | 0.2783 | 14.99 | <0.0001 |
| Joint Stiffness | -10.42 | 18.89 | -21.00 | 18.35 | -32.24 | 18.84 | 0.0003 | 0.0190 | 17.62 | <0.0001 |
| Rest | -5.00 | 23.36 | -19.62 | 25.50 | -34.26 | 26.99 | 0.0001 | 0.0218 | 18.40 | <0.0001 |
| Sleep | -5.48 | 18.74 | -16.67 | 19.53 | -24.07 | 18.32 | 0.0002 | 0.2289 | 14.37 | <0.0001 |
| Impact* | -4.86 | 15.80 | -16.88 | 17.15 | -25.56 | 13.82 | <0.0001 | 0.0446 | 23.95 | <0.0001 |

*Scale is a component of the 3-factor structure and brings together items from the Energy/Tiredness (2 items), Rest (1 item) and Sleep (2 items) scales.

## Supplementary Table 9: Participant demographics and characteristics for the concept elicitation interviews

| **Characteristic** | **Concept elicitation interviews (N=30)** |
| --- | --- |
| Age, mean (range) | 53.4 (21–80) |
| Female, n (%) | 19 (63) |
| Length of time RA symptoms in months, mean (range) | 131.5 (12–600) |
| Race, n (%)  White  Black/African American  Hispanic | 17 (57)  10 (33)  3 (10) |
| Ethnicity, n (%)  Hispanic or Latino (of any race)  Non-Hispanic or Latino | 3 (10)  27 (90) |
| Living Status, n (%)  Living with husband/wife/partner  Living alone  Living with parents  Living with your children  Living with other family members  Living with friends | 15 (50)  9 (30)  3 (10)  1 (3)  1 (3)  1 (3) |
| Highest level of education, n (%)  High school diploma or GED  Graduate or professional degree  Some years of college  College or University degree (2 or 4 year)  Some high school | 12 (40)  7 (23)  6 (20)  3 (10)  2 (7) |
| Work Status, n (%)  Working full-time (in paid employment)  Retired  Unable to work due to rheumatoid arthritis  Unemployed  Working part-time (in paid employment)  Other | 11 (37)  7 (23)  7 (23)  2 (7)  2 (7)  1 (3) |
| Currently receiving disability/health benefits due to RA, n (%) | 9 (30) |
| How would you rate your health in general?  n (%)  Excellent  Good  Fair  Poor | 4 (13)  14 (47)  11 (37)  1 (3) |
| Number of days of morning stiffness over the past 7 days  0 days, n (%)  2 days, n (%)  3 days, n (%)  4 days, n (%)  5 days, n (%)  7 days, n (%)  Other (4–5 days) | 1 (3)  1 (3)  1 (3)  4 (13)  3 (10)  19 (63)  1 (3) |
| Average length of morning stiffness (minutes) over the past 7 days, mean (range) | 187.7 (0–1520) |
| Rating of morning stiffness over past 7 days (scale of 0–10), mean (range)  Frequencies of each response, n (%)  0  3  4  5  6  7  8  9  10 | 6.3 (0–10)  1 (3)  1 (3)  1 (3)  10 (33)  3 (10)  5 (17)  4 (13)  3 (10)  2 (7) |

GED, general education diploma; RA, rheumatoid arthritis; SD, standard deviation

1. Hochberg, M.C., et al., *The American College of Rheumatology 1991 revised criteria for the classification of global functional status in rheumatoid arthritis.* Arthritis Rheum, 1992. **35**(5): p. 498-502.
